# Progression of Beat-to-Beat Blood Pressure Variability Despite Best Medical Management

**DOI:** 10.1161/HYPERTENSIONAHA.120.16290

**Published:** 2020-11-30

**Authors:** Alastair J.S. Webb, Amy Lawson, Karolina Wartolowska, Sara Mazzucco, Peter M. Rothwell

**Affiliations:** From the Wolfson Centre for Prevention of Stroke and Dementia, University of Oxford, United Kingdom.

**Keywords:** blood pressure, hypertension, linear models, prognosis, risk factors

## Abstract

Beat-to-beat variability in blood pressure (BP) is associated with recurrent stroke despite good control of hypertension. However, no study has identified rates of progression of beat-to-beat BP variability (BPV), its determinants, or which patient groups are particularly affected, limiting understanding of its potential as a treatment target. In consecutive patients one month after a transient ischaemic attack or nondisabling stroke (Oxford Vascular Study), continuous noninvasive BP was measured beat-to-beat over 5 minutes (Finometer). Arterial stiffness was measured by carotid-femoral pulse wave velocity (Sphygmocor). Repeat assessments were performed at the 5-year follow-up visit and agreement determined by intraclass correlation coefficient. Rates of progression of systolic BPV (SBPV) and diastolic BPV (DBPV) and their determinants were estimated by mixed-effect linear models, adjusted for age, sex, and cardiovascular risk factors. One hundred eighty-eight of 310 surviving, eligible patients had repeat assessments after a median of 5.8 years. Pulse wave velocity was highly reproducible but SBPV and DBPV were not (intraclass correlation coefficient: 0.71, 0.10, and 0.16, respectively), however, all 3 progressed significantly (pulse wave velocity, 2.39%, *P*<0.0001; SBPV, 8.36%, *P*<0.0001; DBPV, 9.7, *P*<0.0001). Rate of progression of pulse wave velocity, SBPV, and DBPV all increased significantly with age (*P*<0.0001), with an increasingly positive skew and were particularly associated with female sex (pulse wave velocity *P*=0.00035; SBPV *P*<0.0001; DBPV *P*<0.0001) and aortic mean SBP (SBPV *P*=0.037, DBPV *P*<0.0001). Beat-to-beat BP variability progresses significantly in high-risk patients, particularly in older individuals with elevated aortic systolic pressure. Beat-to-beat BPV and its progression represent potential new therapeutic targets to reduce cardiovascular risk.

## Abstract 2

Supplemental Digital Content is available in the text.

Patients with episodic hypertension after a cerebrovascular event have a high risk of recurrent stroke,^1,2^ residual visit-to-visit variability in blood pressure (BPV) on treatment has a poor prognosis despite good control of mean BP^1,2^ and benefits of some antihypertensive drugs in the prevention of stroke may partly result from reduced variability in systolic blood pressure (SBP).^3,4^ Strong associations between visit-to-visit BP variability with cardiovascular events,^5^ renal impairment,^6^ and cognitive decline^7^ have now been demonstrated in population-based cohorts and specific disease cohorts,^6,8^ with similar predictive value of BP variability day-to-day on home readings.^9^ However, use of both visit-to-visit and home BP variability require a prolonged period of assessment, good patient compliance, and follow-up visits.

Variability in BP from one beat to the next (beat-to-beat, BPV) is also associated with an increased risk of recurrent stroke or cardiovascular events in patients with a transient ischaemic attack (TIA) or minor stroke,^10^ with a similar physiological profile to home day-to-day BPV,^11^ while enabling BPV assessment at a single visit. Despite cross-sectional associations with age, the longitudinal change in beat-to-beat BPV with time has not been assessed, and the factors that determine progression of BPV are unknown. To further assess the potential clinical utility of beat-to-beat BPV as a disease marker it is necessary to understand the rate of progression and its determinants to identify potential treatment targets.

Therefore, in a prospective cohort of patients with TIA or minor stroke, we determined the rate of progression of beat-to-beat BPV and identified determinants of greater progression.

## Methods

### TOP Statement

The data that support the findings of this study are available from Professor Rothwell (peter.rothwell@ndcn.ox.ac.uk) upon reasonable request.

### Study Population

Consecutive, consenting patients with TIA or minor stroke were recruited between September 2010 and September 2019, as part of the Phenotyped Cohort of the OXVASC (Oxford Vascular Study). Participants were recruited at the OXVASC daily emergency assessment clinic, following a referral after attendance at the Emergency Department or from primary care, usually within 24 hours. Patients were referred after transient neurological symptoms or symptoms consistent with a minor stroke, not requiring direct admission to hospital. The OXVASC population consists of >92 000 individuals registered with about 100 primary-care physicians in Oxfordshire, United Kingdom. All consenting patients underwent a standardized medical history and examination, ECG, blood tests, and a stroke protocol magnetic resonance imaging brain and contrast-enhanced magnetic resonance angiography (or CT-brain and carotid Doppler ultrasound or CT-angiogram), an echocardiogram and 5-day ambulatory *R* test cardiac monitor. All patients were assessed by a study physician, reviewed by the senior study neurologist (P.M. Rothwell), and are followed-up face-to-face at 1, 3, 6, and 12 months and 2, 5, and 10 years. Medication is prescribed according to guidelines, most commonly with dual antiplatelets (aspirin and clopidogrel) for one month, high dose statins (atorvastatin, 40–80 mg), and a combination of perindopril and indapamide, with the addition of amlodipine as required, to reach a target of <130/80, guided by home telemetric BP monitoring for the first month in the majority of participants.

As part of the OXVASC Phenotyped cohort, a routine prospective cardiovascular physiological assessment is performed at the 1-month follow-up visit. Since August 2017, all surviving participants still registered with OXVASC primary-care physicians are eligible to undergo a repeat physiological assessment when attending for their 5-year follow-up visit. Participants undergoing a repeat study as part of an assessment for a recurrent cerebrovascular event >2.5 years after their initial physiological assessment could also be included. Participants were excluded if they were under 18 years, cognitively impaired (mini-mental state examination <23), pregnant, had autonomic failure, a recent myocardial infarction, unstable angina, heart failure (New York Heart Association, 3–4 or ejection fraction <40%), or untreated bilateral carotid stenosis (>70%). OXVASC is approved by the Oxfordshire Research Ethics Committee A.

Beat-to-beat BPV was measured after at least 15 minutes supine rest over 5 minutes in a quiet, temperature-controlled room (21–23 °C). Continuous ECG and noninvasive BP were acquired at 200 Hz (Finometer, Finapres Medical Systems, the Netherlands), via a Powerlab 8/30 with LabChart Pro software (ADInstruments). Waveforms were preferentially recorded from the middle phalanx of the middle finger. Automated calibration (Physiocal) was performed until the recording was stable, but turned off during each test. Brachial waveforms (Finometer) were derived from finger pressures and calibrated offline by linear regression to 2 to 3 supine, oscillometric brachial readings on the contralateral arm, performed immediately before the monitoring period, with manual exclusion of artefacts. In patients with a significant deterioration in recording quality during the first 5 minutes, the test was stopped, and the calibration procedure repeated. If necessary, the finger cuff was moved to an adjacent finger or the proximal phalanx of the same finger, or the hand was warmed with a hand warmer. Before physiological assessment, 2 sitting clinic BPs, 5 minutes apart, were measured at ascertainment and 1 month in the nondominant arm, by trained personnel. Applanation tonometry (Sphygmocor, AtCor Medical, Sydney, Australia) was used to measure carotid-femoral pulse wave velocity (aortic-PWV), aortic augmentation index, and aortic SBP and diastolic blood pressure (DBP) and pulse pressure.

Consistency in measures between baseline and follow-up were determined by intraclass correlation coefficients and linear regression, and visually by Bland-Altman plots. Significant changes in indices between baseline and follow-up were assessed by paired *t* test. Rates of progression of measures of arterial stiffness, aortic BP, and cerebral blood flow velocity were determined by linear mixed-effect models, with autoregressive covariance to account for repeated measures. Rates of progression were determined by the interaction with the time interval between visits, as continuous indices and stratified by age group (<55, 55–65, 65–75, >75). Potential determinants of absolute values and rates of progression were assessed, unadjusted, and adjusted for age, gender, and cardiovascular risk factors (smoking, dyslipidemia, diabetes, hypertension).

Analyses were performed in R and Matlab r2018, using in-house software.

## Results

One hundred eighty-eight of 310 eligible, surviving patients were reviewed at a median of 5.8 years after the initial assessment. Of the included patients, 150 had arterial stiffness assessments at baseline and follow-up, while 170 had beat-to-beat BPV. Demographic characteristics were similar between patients undergoing arterial stiffness and beat-to-beat BPV (Table 1), and between patients having any repeat study versus those eligible surviving patients who did not return for follow-up (Table S1 in the Data Supplement).

**Table 1.**
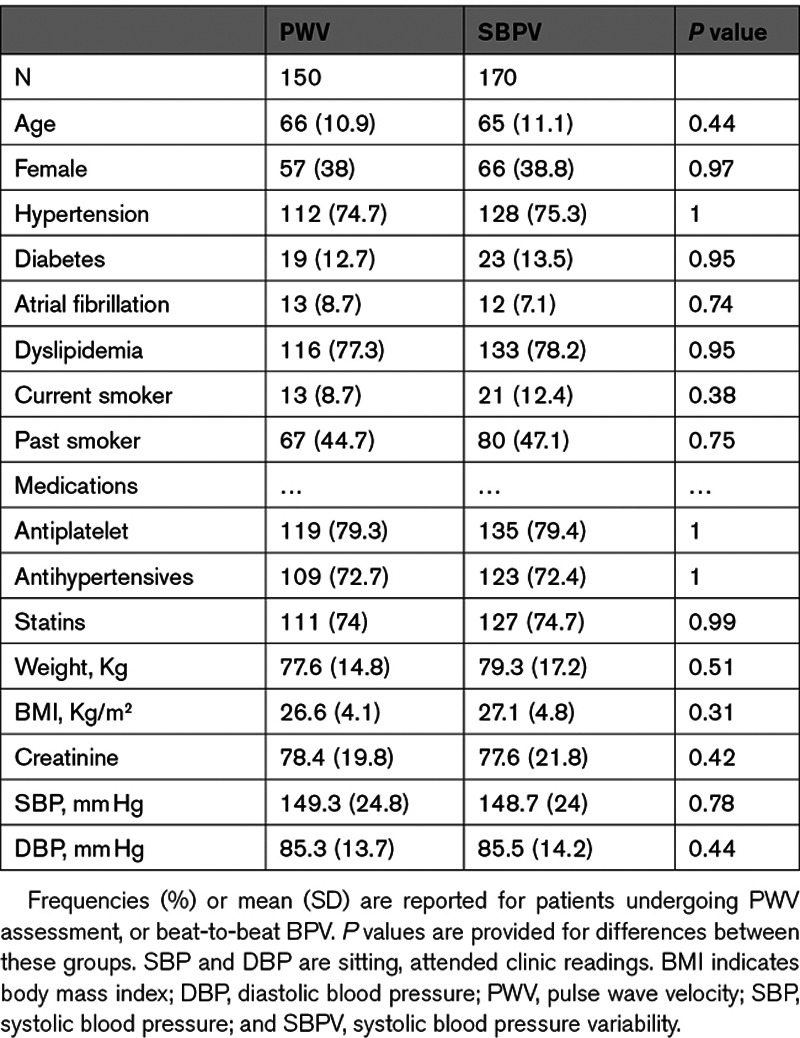
Baseline Characteristics of Patients With Follow-Up Studies

**Figure 1.**
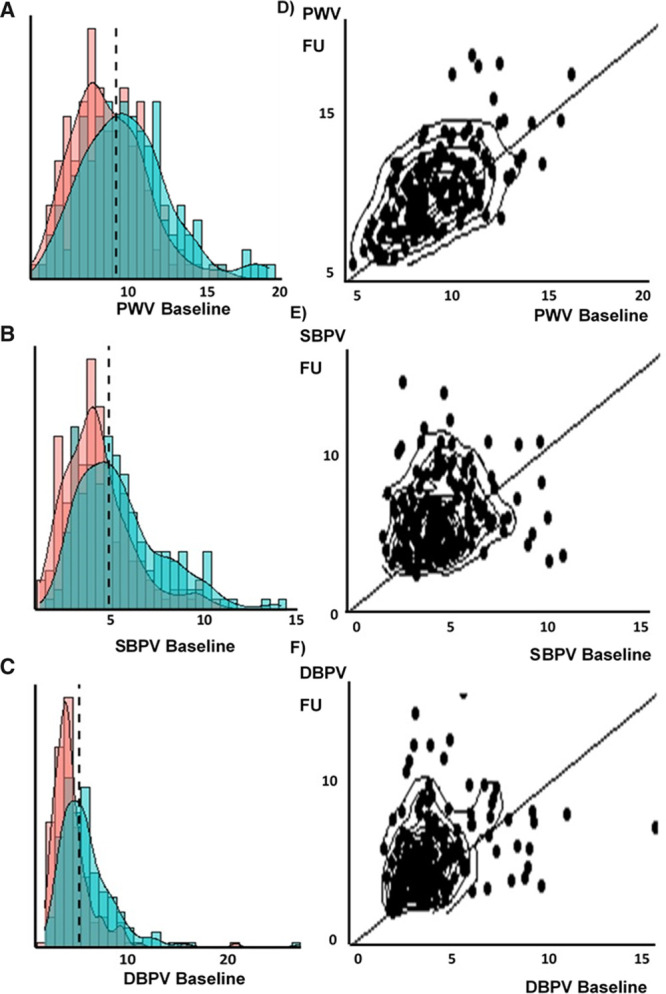
**Change in distribution of arterial stiffness and blood pressure variability between baseline and follow-up.** **A–C**, The distribution of each index at baseline (red) and follow-up (blue). **D–F**, The correlation between baseline and follow-up as scatter plots with contour lines and lines of unity. DBPV indicates diastolic blood pressure variability; PWV, pulse wave velocity; and SBPV, systolic blood pressure variability.

Arterial stiffness (PWV) was highly reproducible within individuals over the 5 years of follow-up (Table 2), but SBPV and DBPV were only weakly reproducible. However, there was a significant mean increase in PWV, SBPV, and DBPV, with a greater positive skew of the distribution for SBPV and DBPV at follow-up (Figure 1). In contrast, heart rate variability and baroreceptor sensitivity (BRS) were more reproducible than beat-to-beat BP variability, but there was no significant progression of heart rate variability during follow-up, with a marginal fall in BRS (Table 2).

**Table 2.**
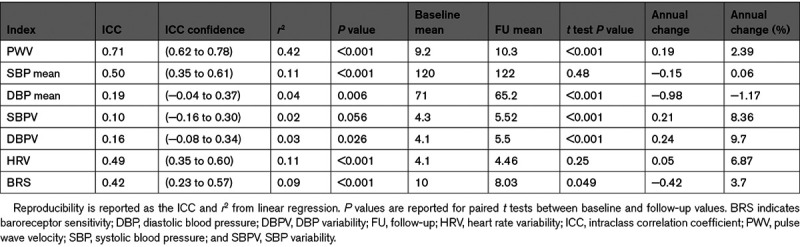
Reproducibility and Progression of Arterial Stiffness, Aortic Blood Pressure, and Blood Pressure Variability

Cross-sectionally, BPV was associated with greater age at follow-up, more evident in women than men (Figure S1), but there was no cross-sectional association between age and SBPV at baseline (*P*=0.56). This reflected greater attrition of patients with higher baseline SBPV or arterial stiffness, with both SBPV and PWV predicting loss to follow-up, even after adjustment for age and gender (SBPV *P*=0.048; PWV *P*=0.021).

**Figure 2.**
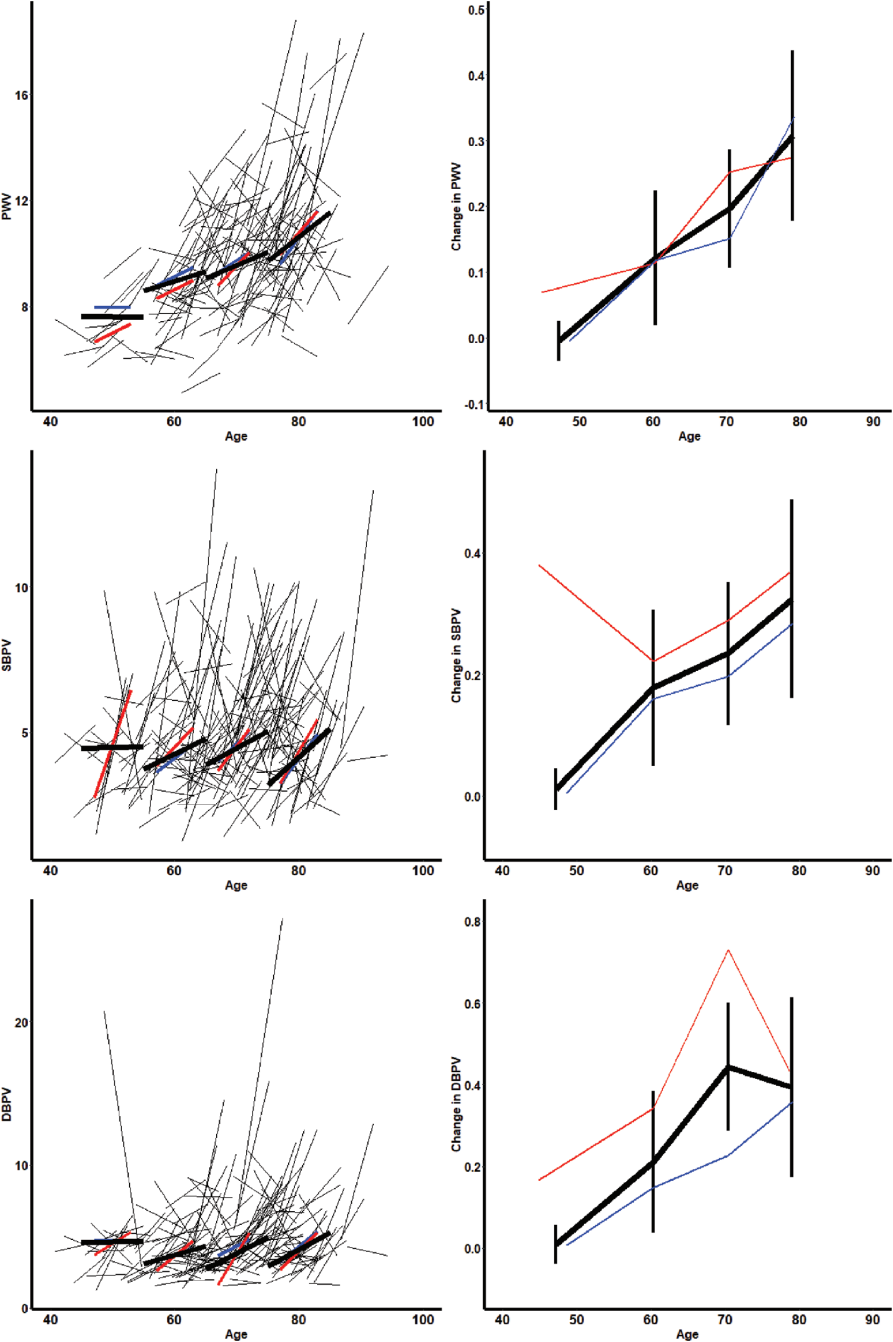
**Progression of arterial stiffness and blood pressure variability by age and gender.** **A–C**, Individual changes during follow-up, and summary estimates within age groups (<55, 55–65,65–75, >75), for all patients (black), for men (blue), and women (red). **D–F**, The average rate of progression within each age group, stratified by age, and gender, with 95% CIs for the whole population. DBPV indicates diastolic blood pressure variability; PWV, pulse wave velocity; and SBPV, systolic blood pressure variability.

In mixed-effect linear models, PWV was greater in older patients and increased at a greater rate at older ages (Figure 2). Similarly, the rate of increase for SBPV and DBPV increased significantly with increasing age and female gender (Table 3), although due to the lower reproducibility of BPV and the greater attrition of older patients with greater SBPV at baseline, there was no cross-sectional association between age and SBPV (Figure 2). In contrast, there was no significant progression in mean aortic SBP and a consistent small fall in aortic DBP across ages (Figure S3), although the rate of change of mean DBP did not increase with increasing age. A history of diabetes was associated with both a greater cross-sectional PWV and a greater rate of progression of PWV, but did not predict increased SBPV, DBPV, or their progression. However, other demographic indices including AF, smoking, and weight were associated with greater BPV cross-sectionally but a decrease in BPV with time, likely reflecting regression to the mean or attrition bias.

**Table 3.**
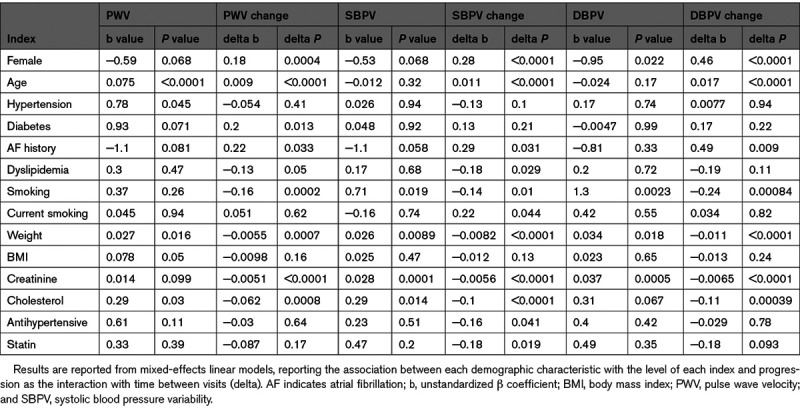
Association Between Demographic Characteristics and Progression of Key Indices of Arterial Stiffness and Blood Pressure Variability

Absolute SBPV and DBPV level were not associated with PWV after adjustment for age, gender, and risk factors, but increased PWV was associated with progression of DBPV (Table 4) and a fall in mean DBP (Table S4) with time. Greater heart rate variability was associated with greater BPV level but not with progression of BPV over time. In contrast, a lower BRS was associated with increased BPV, while a greater BRS was associated with greater progression of BPV over time, although this may reflect regression to the mean.

**Table 4.**
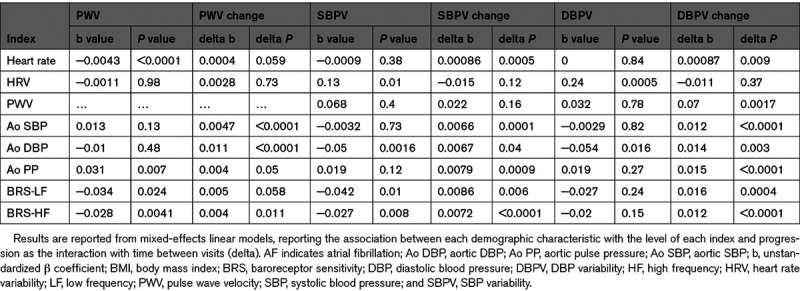
Association Between Physiological Characteristics and Progression of Key Indices of Arterial Stiffness and Blood Pressure Variability

Progression of both SBPV and DBPV were most strongly associated with an elevated aortic SBP and DBP, and particularly with an increased aortic pulsatility, both before (Table S4) and after (Table S5) adjustment for age and gender. Even after further adjustment for cardiovascular risk factors, PWV and the other aortic measure, progression of SBPV and DBPV were still associated with an elevated aortic SBP (SBPV *P*=0.037, DBPV *P*<0.001) but not with aortic DBP (SBPV *P*=0.53, DBPV *P*=0.15).

## Discussion

In this first study of progression of beat-to-beat variability, SBP and DBP variability progressed at a greater rate with increasing age, female gender, and a greater aortic BP. Furthermore, there was a greater positive skew of SBPV and DBPV at follow-up compared with baseline. This paralleled the greater rate of increase in arterial stiffness with age but PWV only predicted an increase in DBP variability, despite both PWV and SBPV predicting a greater loss to follow-up. Overall, aortic SBP was particularly associated with an increase in SBPV or DBPV, after adjustment for demographic factors, arterial stiffness, and DBP.

Visit-to-visit,^2^ day-to-day,^9,12^ and diurnal BP variability^13,14^ predict the risk of stroke and future cardiovascular events, independent of mean BP. Calcium channel blockers and thiazide-like diuretics reduce BPV,^3,4,15^ with an associated difference in the rate of recurrent cardiovascular events. However, these methods require repeated assessments and the magnitude of the prognostic value of BPV compared with mean BP alone is uncertain, limiting their current use in clinical practice.^16^ Beat-to-beat blood pressure variability can be assessed in a single clinic visit, is cross-sectionally associated with clinical markers of cardiovascular risk, is associated with similar underlying physiological processes as day-to-day BP variability^11^ and predicts outcome after acute stroke^17^ and the risk of recurrent stroke and cardiovascular events.^10^ This study now demonstrates that beat-to-beat BPV progresses significantly over time despite excellent risk factor control, predicts loss to follow-up, and that this progression parallels progression of arterial stiffness. Similar data was not available to assess the progression of other forms of BPV, but the progression of beat-to-beat BPV supports the potential of beat-to-beat BPV as a direct treatment target. However, the association with SBP also raises the possibility that even more intensive BP control to levels suggested in the Systolic Blood Pressure Intervention Trial (SPRINT) trial^18^ may further limit progression of arterial disease through limiting progression of BPV. Furthermore, BPV increased quicker with age with a greater positive skew, potentially identifying that the risk of recurrent events is particularly related to BPV in more elderly patients. The association of progression with aortic pulsatility is consistent with their common cause as markers of vascular aging, but the strong progression of BPV over time suggests it is likely to be have independent prognostic significance and may represent an additional treatment target.

Despite similar physiological and demographic associations,^11^ beat-to-beat BPV measures BPV over a much shorter time period than other forms of BPV. Therefore, although beat-to-beat BPV may vary from day-to-day or season to season, a beat-to-beat BPV value does not directly measure diurnal or seasonal variability,^19^ adherence to medication^20^ or day-to-day environmental factors such as weekday versus weekend readings,^21^ unlike longer forms of BPV. Instead, it predominantly reflects current autonomic function^22^ and is the primary input in determining resting-state BRS and dynamic cerebral autoregulation.^23^ It has the potential, therefore, to more directly measure loss of compensatory mechanisms in older patients and is a key determinant of the short-term gain of both baroreceptor function and cerebral autoregulation. However, given its similar associations with longer forms of BPV, it may still provide a more efficient method of determining the same prognostic information. Alternatively, it may provide different or additional information and be affected by alternative treatment options, including mechanical^24^ and pharmacological interventions.^25^

This study does have some limitations. It is still relatively small given the high rate of attrition in a high-risk population, but it is the first study to assess beat-to-beat BP variability at an interval of 5 years and, despite the moderate size, associations were strong and consistent with previous evidence. Second, due to the age and frailty of the population, only a proportion of the baseline population could be reassessed at 5 years with BPV at baseline predicting greater attrition at follow-up, thus resulting in a likely conservative underestimation of the cross-sectional and longitudinal associations between age and BPV. This rate of attrition also implies potential prognostic significance of BPV. However, this level of attrition increases the probability of bias, although any bias is likely to be conservative, and limits the applicabilty of the results to primary prevention populations. Ideally, this study will need replicating in these groups. Third, due to the low reproducibility of BPV, there was evidence of regression to the mean, with an opposite direction of association with the absolute level of BPV and its progression for many risk factors. However, despite this, regression to the mean was not evident for age, gender, PWV, and aortic BP, the most important predictors of progression of BPV. Fourth, the low reproducibility of BPV may limit its utility in monitoring response to treatment and its validity as a measure of BPV in a single visit. However, as this study only looked at 5-year follow-up, further research is required to assess the short-term reproducibility of BPV. Furthermore, development of protocols to measure elevated BPV at one or more assessments may still identify patients at an increased risk and invoke a change in management while the significance of elevated beat-to-beat BPV provides insights into potential novel physiological mechanisms and new treatment targets. Finally, we have not shown that progression of BPV itself predicts the risk of recurrent cardiovascular events, or that it is amenable to treatment.

This study demonstrates that beat-to-beat BPV progresses significantly over 5 years, and that this is predicted by a higher aortic SBP and pulsatility, after adjustment for age, gender, and risk factors. However, this is the first large study to report these associations, and it is important to replicate this in other populations. Furthermore, proof of a causative role for BPV will require evidence of an association between progression of BPV and future cardiovascular risk, and identify whether this association is particularly important for specific stroke causes, such as cerebral small vessel disease. In addition, further research is required to understand the relationship between beat-to-beat and other forms of BPV, and the role of historic hypertension in driving BPV and its progression.The potential causative relationship between aortic SBP and BPV also requires evidence from randomized trials that consistent control of BP prevents progression of beat-to-beat BPV over time, and that this mediates some of the subsequent reduction in cardiovascular events. Finally, it is vital to identify interventions that reduce BPV and determine whether control of BPV independent of control of mean BP reduces the risk of future cardiovascular events.

In conclusion, beat-to-beat BP variability progresses over 5 years in high-risk patients with recent TIA or minor stroke, with higher rates of progression in older patients and patients with higher aortic BPs. Despite excellent risk factor control, better control of aortic SBP may prevent progression of SBPV and reduce risk of recurrent events. Older patients are most likely to be at an increased risk due to progression of BPV, and therefore, interventions to reduce beat-to-beat BPV may best be targeted to these patients.

## Perspectives

Beat-to-beat BP variability is associated with an increased risk of recurrent stroke after TIA or minor stroke, independent of mean BP, and may provide a novel treatment target to reduce future cardiovascular risk. The demonstration that it progresses significantly over 5 years, and that this progression is associated with higher aortic SBP and age, suggests that it may be one mechanism mediating the relationship between long-term hypertension and cardiovascular outcomes. If so, good control of hypertension may prevent its progression, while interventions to directly reduce beat-to-beat BPV may reduce the residual risk of cardiovascular events that persist despite control of hypertension. However, further studies are required to determine whether beat-to-beat BPV and its progression provides significant additional prognostic information, whether this applies to other populations and whether this differs from the prognostic significance of other forms of BPV. Furthermore, clinical trials are required to identify treatments to reduce beat-to-beat BPV and its progression, and to determine whether these are associated with a reduction in subsequent clinical events.

## Acknowledgments

We are grateful to all the staff in the general practices that collaborated in the Oxford Vascular Study: Abingdon Surgery, Stert St, Abingdon; Malthouse Surgery, Abingdon; Marcham Road Family Health Centre, Abingdon; The Health Centre, Berinsfield; Key Medical Practice; Kidlington; 19 Beaumont St, Oxford; East Oxford Health Centre, Oxford; Church Street Practice, Wantage. This work uses data provided by patients and collected by the National Health Service as part of their care and support and would not have been possible without access to this data. The National Institute for Health Research recognizes and values the role of patient data, securely accessed and stored, both in underpinning and leading to improvements in research and care.

## Sources of Funding

The Oxford Vascular Study is funded by the National Institute for Health Research (NIHR) Oxford Biomedical Research Centre (BRC), Wellcome Trust, Wolfson Foundation, British Heart Foundation and the European Union’s Horizon 2020 programme (grant 666881, SVDs@target). P.M. Rothwell is in receipt of a NIHR Senior Investigator award. A.J.S. Webb and his work is funded by a Wellcome Trust Clinical Research Development Fellowship (206589/Z/17/Z) and British Heart Foundation Project Grant (PG/16/38/32080). The views expressed are those of the authors and not necessarily those of the National Health Service, the NIHR or the Department of Health.

## Disclosures

None.

## Supplementary Material



## Novelty and Significance

### What Is New?

- Beat-to-beat blood pressure variability (BPV) increases significantly over 5 years despite intensive risk factor control.
- Beat-to-beat BPV becomes more positively skewed with age and time.
- Progression of BPV is associated with age, female gender, and aortic systolic BP.

### What Is Relevant?

- Older patients are most likely to have a marked increase in BPV.
- Rigorous control of BP may help to prevent progression of BPV.
- Control of BPV and its prevention may be a novel treatment target.

### Summary

Beat-to-beat BPV and its progression may be a new therapeutic target to reduce cardiovascular risk.
