# Supplementary figures and images for: Progression of Beat-to-Beat Blood Pressure Variability Despite Best Medical Management
Source: Hypertension. 2020 Nov 30;77(1):193–201. doi: 10.1161/HYPERTENSIONAHA.120.16290 (PMC7720874; doi:10.1161/HYPERTENSIONAHA.120.16290)

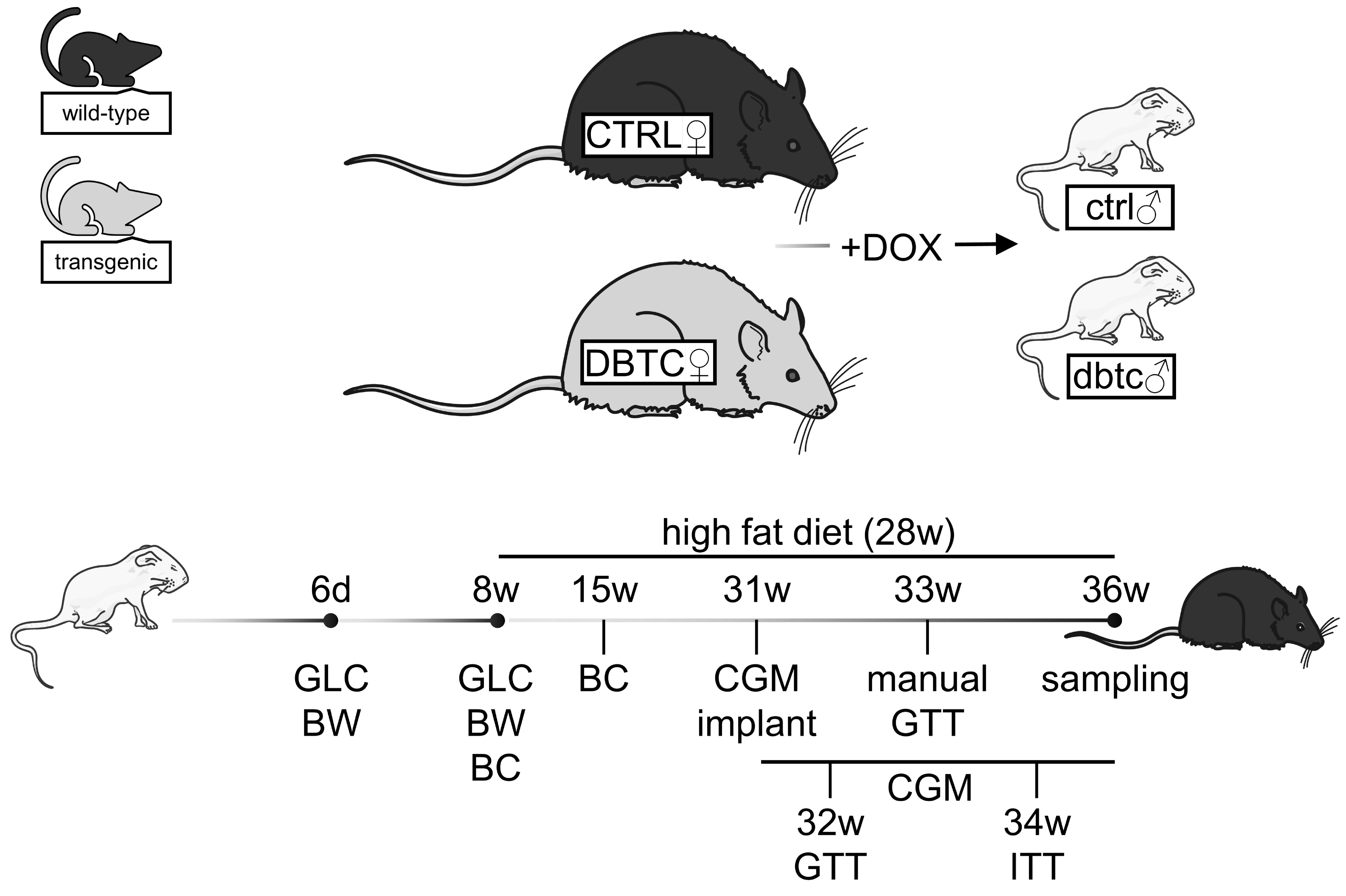

Supplement: Supplementary file 1 [file hyp-77-193-s001.jpg]
