# Supplementary material for: Progression of Beat-to-Beat Blood Pressure Variability Despite Best Medical Management
Source: Hypertension. 2020 Nov 30;77(1):193–201. doi: 10.1161/HYPERTENSIONAHA.120.16290 (PMC7720874; doi:10.1161/HYPERTENSIONAHA.120.16290)
Supplement: Supplementary file 2 [file hyp-77-193-s002.doc]

# Intrauterine exposure to diabetic milieu does not induce diabetes and obesity in male adulthood in a novel rat model

Till Schütte ^1,2,3,4^, Sarah Kedziora ^1,3,4,5,6^, Nadine Haase ^1,3,4,5,6^, Florian Herse ^1,3,5,6^, Andreas Busjahn ^8^, Anna Birukov ^4,6,9,10^, Natalia Alenina ^3,4,5^, Dominik N. Müller ^1,3,4,5,6^, Michael Bader ^1,3,4,5,7^, Michael Schupp ^1,2,3^, Ralf Dechend ^1,3,4,5,6,11^, Kristin Kräker* ^1,3,4,5,6^, Michaela Golic* ^1,3,4,5,6^

* shared authorship

1. Charité – Universitätsmedizin Berlin, Corporate Member of Freie Universität Berlin, Humboldt‑Universität zu Berlin, and Berlin Institute of Health, Germany.
2. Institute of Pharmacology, 10115 Berlin, Germany.
3. Berlin Institute of Health (BIH), 10178 Berlin, Germany.
4. DZHK (German Center for Cardiovascular Research), Partner Site Berlin, Berlin, Germany.
5. Max Delbrück Center for Molecular Medicine in the Helmholtz Association, Berlin, Germany.
6. Experimental and Clinical Research Center – a joint cooperation between the Max‑Delbrück‑Center for Molecular Medicine and the Charité – Universitätsmedizin Berlin, Germany.
7. Institute for Biology, University of Lübeck, Lübeck, Germany.
8. HealthTwiSt GmbH, Berlin, Germany.
9. Department of Molecular Epidemiology, German Institute of Human Nutrition Potsdam-Rehbrücke, Nuthetal, Germany
10. German Center for Diabetes Research, München-Neuherberg, Germany
11. HELIOS‑Klinikum, Department of Cardiology and Nephrology, Berlin, Germany.

## Supplemental Table S1. Glycemic control characteristics during metabolic tolerance tests deduced from telemetric device.

There were no differences in area under the curve (AUC), glucose peak/nadir, time to peak/nadir, and delta glucose after glucose or insulin injection, independent of dietary challenge. Male WT offspring of normoglycemic (ctrl control, normal chow/high fat diet, n(GTT) 6/7, n(ITT) 6/8) and diabetic mothers (dbtc diabetic, normal chow/high fat diet, n(GTT) 7/7, n(ITT) 7/8), intraperitoneal glucose tolerance test (i.p. GTT), subcutaneous insulin tolerance test (s.c. ITT), unpaired test, mean±SEM, ns not significant.

## Supplemental Figure S1. Exemplarily trend, diurnal rhythm, and noise analysis conducted by CGM of male WT offspring of normoglycemic pregnancy fed a normal chow.

## Supplemental Figure S2. Exemplarily trend, diurnal rhythm, and noise analysis conducted by CGM of male WT offspring of diabetic pregnancy fed a normal chow.

## Supplemental Figure S3. Exemplarily trend, diurnal rhythm, and noise analysis conducted by CGM of male WT offspring of normoglycemic pregnancy fed a high fat diet.

## Supplemental Figure S4. Exemplarily trend, diurnal rhythm, and noise analysis conducted by CGM of male WT offspring of diabetic pregnancy fed a high fat diet.

## Supplemental Figure S5. Maternal C-peptide level at the end of pregnancy.

C‑peptide level of transgenic Tet29 females was elevated on day 22 compared to control pregnancy. Maternal wild-type females (CTRL control, n 12) and transgenic Tet29 females (DBTC diabetic, n 14), mean±SEM, unpaired test, **p<0.01.

## Supplemental Figure S6. Measurements of body weight and composition before and during dietary challenge.

Body weight and body composition were unaltered regarding absolute body weight **(A)**, free water **(B)**, fat mass **(C)** and muscle mass **(D)** between offspring of diabetic compared to control pregnancy before (8 weeks of age) and during dietary challenge (15 weeks of age). By cross comparing the diets contributing to the absolute body weight, the animals fed a high fat diet were significantly heavier. In addition, they were composed of more fat and less of water and muscle. Male wild-type offspring of normoglycemic (ctrl control, before/normal chow/high fat diet, n 16/8/8) and diabetic mothers (dbtc diabetic, before/normal chow/high fat diet, n 16/8/8), unpaired test, mean±SEM, ns not significant, *p<0.05, **p<0.01, ***p<0.001.

## Supplemental Table S1

**
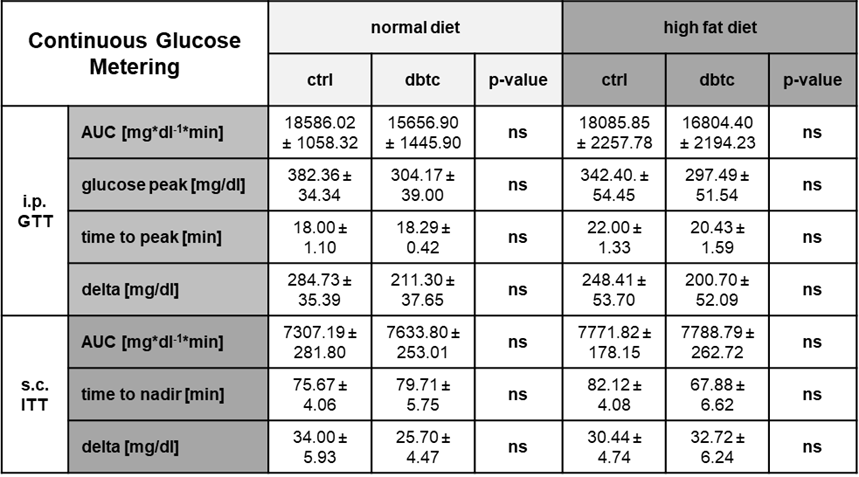
**

## Supplemental Figure S1


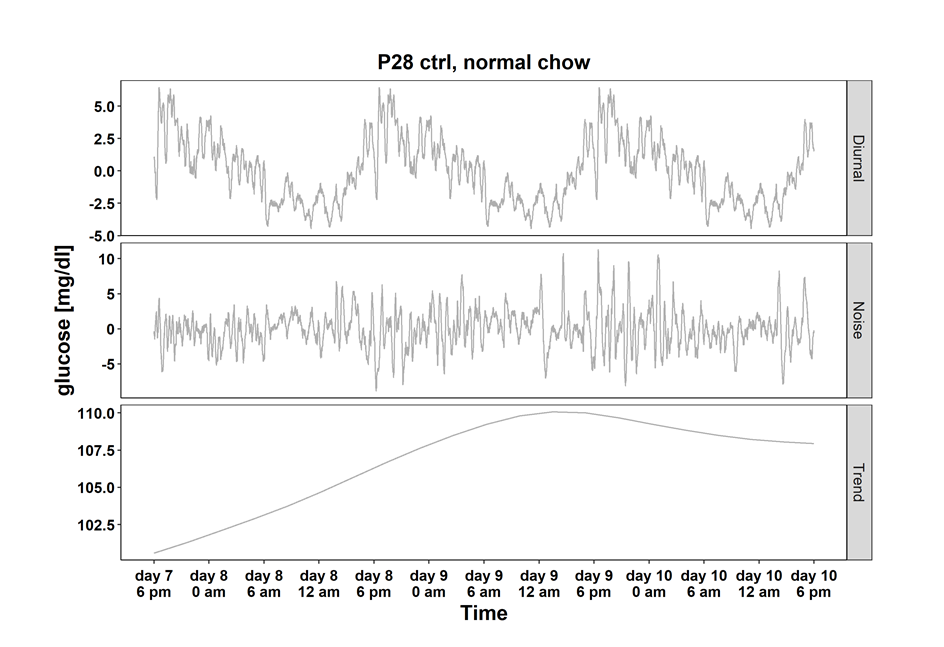


## Supplemental Figure S2


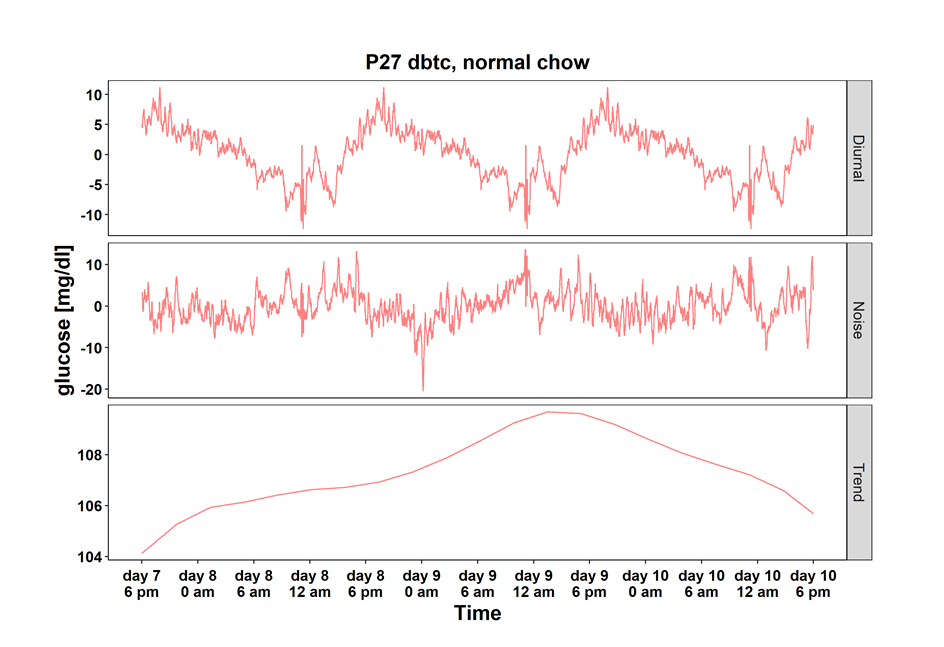


## Supplemental Figure S3


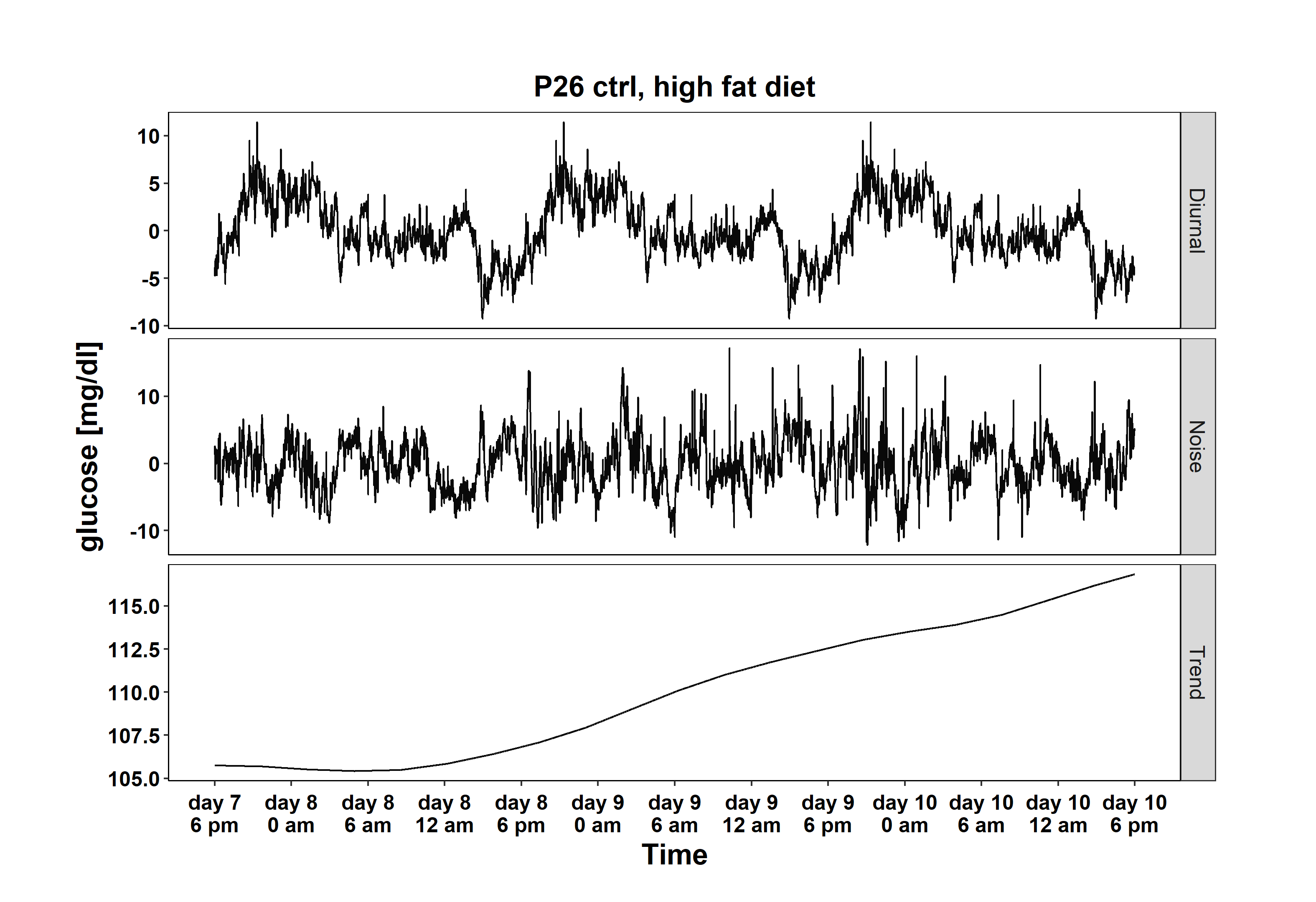


## Supplemental Figure S4


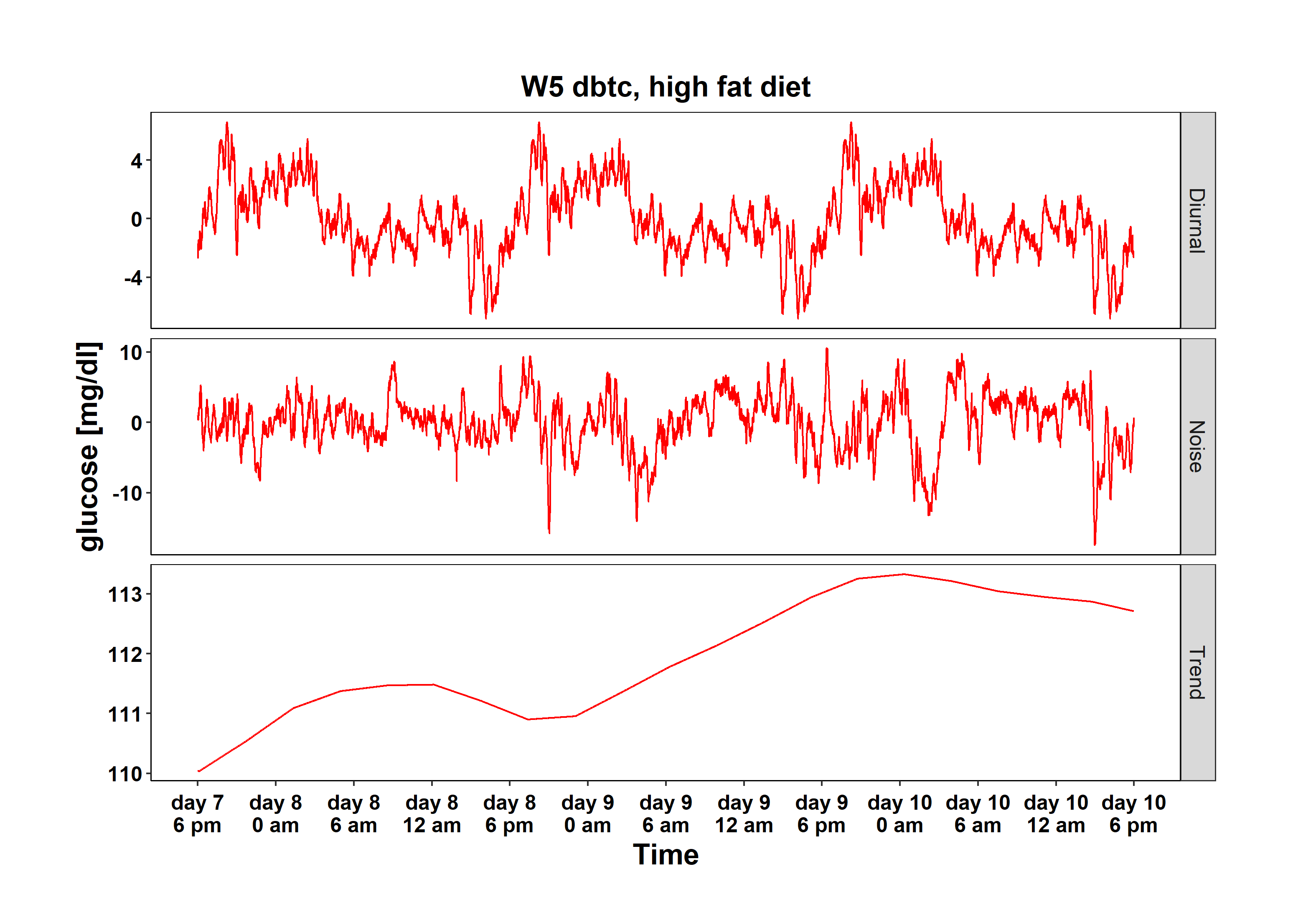


## Supplemental Figure S5


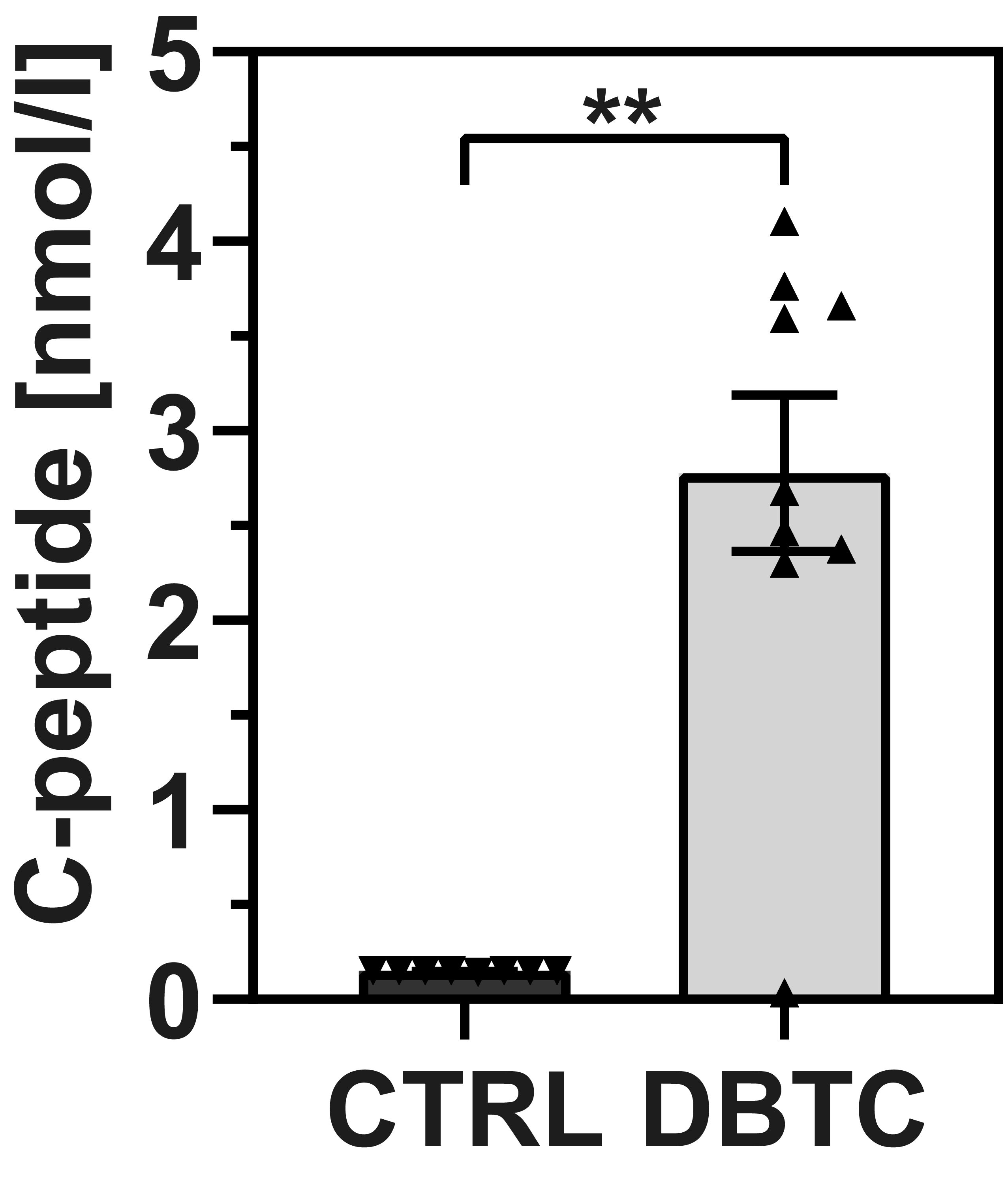


## Supplemental Figure S6

**

**
