# Supplementary material for: Progression of Beat-to-Beat Blood Pressure Variability Despite Best Medical Management
Source: Hypertension. 2020 Nov 30;77(1):193–201. doi: 10.1161/HYPERTENSIONAHA.120.16290 (PMC7720874; doi:10.1161/HYPERTENSIONAHA.120.16290)
Supplement: Supplementary file 3 [file hyp-77-193-s003.pdf]

*** Short In Vivo Checklist**
AHA - Preclinical Animal Testing: Prevention of bias is important for experimental cardiovascular research. ***This short checklist must be completed, and the answers should be clearly presented in the manuscript as well.*** *The checklist will be used by reviewers and editors but will not be published. If a revision is requested, you will be required to complete at revision submission a more detailed checklist that will be published with the accepted article.*

This study involves animals:
Yes

**Animals**	
Species, age, sex, strains, and sources of animals are described:	Yes
**Randomization**	
Randomization and allocation concealment were performed:	Yes
**Blinding**	
Blinding was performed:	Yes
**Inclusions and Exclusions (a)**	
Specific criteria for inclusions and exclusions are specified:	Yes
**Inclusions and Exclusions (b)**	
Criteria for inclusions and exclusions were set before the study:	Yes
**Reporting of Excluded Animals**	
All animals excluded after the randomization are reported:	Yes
**Statistical Methods**	
Statistical Methods are described:	Yes

Date completed: 10/08/2020 12:40:35
User pid: 11242
